# The Prevention of Epidemic Cholera

**Published:** 1865-11

**Authors:** S. S. Salisbury

**Affiliations:** Tolono, Ills.


### THE PREVENTION OF EPIDEMIC CHOLERA.

By S, S. SALISBURY, M. D., of Tolono, Ills.

Epidemic Cholera, being again prevalent, and there being as yet no means of prevention known: I will submit the following facts for the consideration of the medical public.

It is an established fact, that Arsenic, if given in small and repeated doses, may have its poisonous operation averted by keeping the kidneys in active operation at the same time. The close resemblance between the action of arsenic and antimony, and the poison that produces epidemic cholera, leads me to the conclusion that the poison that produces epidemic cholera, must be a material substance, and that its poisonous operation may be averted in the same way.

The poison that produces this disease must necessarily enter the system gradually, whether it be absorbed by the lungs, the skin, or swallowed in the saliva, and at the same time it is gradually passing out of the system by the excretory organs, enabling them to carry off a larger quantity of the poison, and thereby prevent an accumulation of it in the system sufficient to develop the disease, or at least by this means mitigate its violence.

The operation of diluents in preventing strangury when cantharies is absorbed, is a fair example of the idea I wish to convey. In this case we merely, by adding to the quantity of water in the blood, cause the kidneys to excrete a larger quantity of water, which in its passage carries out with it the irritating substance, and prevents its accumulation in the blood.

We know that persons whose excreting organs, and particularly the kidneys, are impaired, which interferes with the proper performance of their function, are more susceptible to the influence of poisons, and this class are more easily affected by cholera and all epidemic and infectious diseases. While on the other hand, those whose excretory organs are in active operation more effectually resist the action of these poisons, and this class of diseases. Although at each visit of cholera we see habitual drunkards and persons with broken down constitutions, that have the functions of their excretory organs impaired, fall thick enough, I think it would be a difficult matter to find a subject of diabetes that had been attacked with cholera.

Cider has been observed to be, to a certain extent, a preventive of epidemic cholera, by the English physicians. The same may be said of our own country. The great fruit country of East Tennessee and Southern Kentucky was affected lightly by the cholera during its last visit to this country, and it was observed by the common people that those who used cider constantly and freely, escaped. This article was manufactured abundantly there, and used as a common drink. That class of ripe fruits and vegetables, the use of which has been thought by some to prevent, rather than provoke an attack, will, on examination, be found to possess diuretic properties.

The following incident has come to my knowledge: During the last visit of epidemic cholera to this country, it prevailed in a small town in Kentucky, and eight or nine young men employed themselves in nursing the sick, and burying the dead; this was at a season when melons were abundant, of which these young men all ate freely the whole time, none of them suffered with any symptom of the disease, although a large proportion of the inhabitants were attacked.

The waters of some of the sulphur springs of this country have attained a degree of celebrity among the people living in their vicinity, for the prevention of cholera. Of these the spring at Delaware, Ohio, and some of the sulphur springs of Kentucky, may be mentioned particularly, and they contain Hydrosulphuric Acid Gas, and may be drank in large quantities without inconvenience, and pass off as rapidly by the kidneys.

The exemption obtained by the use of these articles, has been attributed in one case to the presence of Acid in the blood, and in the other to the presence of Sulphur. They all, however, possess one common property—they are diuretic, either by direct action, or by the quantity taken.

I will suggest the constant use, during the prevalence of the epidemic, of dilutent diuretics, of that class that are neither emetic or cathartic, and of these Mineral (Carbonic Acid) Waters, Acetate of Potass largely diluted, or pure Cream Tartar, in small doses, might be found useful and agreeable. I think the conclusion is a fair one, that if this class of articles was used during the prevalence of the epidemic, it might either be prevented, or at least, its violence mitigated.

